# Mitigating semantic label divergence in federated learning: Obfuscated encoding and alert filtering for security monitoring

**DOI:** 10.1371/journal.pone.0338488

**Published:** 2025-12-29

**Authors:** Yoonho Lee, Joonghyuk Im, Jisu Kim, Myungkeun Yoon

**Affiliations:** Department of Computer Science, Kookmin University, Seoul, South Korea; Alma Mater Studiorum Universita di Bologna: Universita degli Studi di Bologna, ITALY

## Abstract

Federated learning (FL) is emerging as a key approach for collaborative machine learning (ML) in distributed information systems where direct data sharing is infeasible due to policy constraints. In security operations center (SOC) settings, we study FL for the classification of *network intrusion detection system (IDS) alerts*—structured event records emitted by sensors (e.g., Snort/Suricata)—where consistent interpretation of event data is critical for reliable ML-based decision support. However, differences in labeling criteria across organizations often lead to semantic inconsistencies, undermining the accuracy and generalizability of FL models. This paper presents two key contributions that mitigate this issue without requiring raw data exchange. First, we propose *Keyed Feature Hashing (KFH)*, a key-dependent obfuscated encoding scheme that enables consistent vectorization of heterogeneous IDS alerts across entities while reducing the risk of model inversion. Second, we introduce a *filtering mechanism* that leverages KFH representations to identify and exclude alerts likely to be misclassified due to inter-entity label discrepancies. Experiments using a large-scale real-world dataset collected from 14 organizations demonstrate that our method improves classification F1-score by up to 13.36% while maintaining over 99% alert coverage. These contributions enhance the trustworthiness of FL-based decision models in distributed, label-divergent environments.

## Introduction

Federated learning (FL) is becoming a critical paradigm in collaborative information systems, where multiple organizations aim to build machine learning (ML) models without directly sharing sensitive data [1–4]. Especially in domains such as security operations centers (SOCs) [5–8], FL facilitates the construction of globally generalizable models by enabling decentralized training across distributed entities. This is particularly important in scenarios where privacy, regulatory compliance, or competitive concerns prohibit raw data sharing.

Collaborative information systems, such as SOCs, increasingly rely on real-time alert streams to support distributed decision-making. These systems promote cooperation among organizations by enabling the sharing of structured event data, including intrusion detection system (IDS) alerts [5–8]. Such collaboration helps participating entities respond proactively to threats that may recur across institutions. For example, when one organization observes a novel attack pattern from its IDS alerts, early dissemination of this information allows others to prepare countermeasures in advance.

Despite the benefits of collaboration, FL environments introduce new security and consistency challenges. While FL avoids raw data transfer by exchanging only model parameters [1–4], the trained model itself may be leaked or exposed to malicious participants. Since the global model is distributed to a large number of participating entities, the likelihood of model exposure—whether due to insider threats, compromised endpoints, or misconfigurations—is significantly increased. In such cases, adversaries may attempt model inversion attacks to infer sensitive attributes of the training data. To mitigate this risk, it is essential to obfuscate input representations in a way that preserves their utility for training but limits their interpretability when the model is compromised.

A further complication arises from semantic inconsistency of labels across organizations. In distributed environments, alert labels are often assigned based on institution-specific security policies, operational objectives, or contextual constraints. As a result, the same or highly similar alert instance may be labeled as “malicious” by one entity and “benign” by another. We refer to this phenomenon as *inconsistent labeling among different entities* (ILADE). Unlike commonly addressed issues such as class imbalance or non-independent and identically distributed (non-IID) data, ILADE originates from heterogeneous semantic judgments—rather than distributional shifts—and thus requires dedicated mitigation strategies.

For instance, consider an alert triggered by a commonly used network scanning or remote administration tool. One organization may classify such activity as benign if it originates from authorized internal operations, while another may label the same behavior as malicious based on its resemblance to known attack patterns [7]. When such divergent interpretations are aggregated during FL training, the resulting global model is exposed to label conflicts that degrade its learning stability and predictive accuracy.

Existing approaches to FL, such as FedAvg [1] and FedProx [2], primarily aim to address statistical heterogeneity or data imbalance. However, they do not account for semantic misalignments in labeling, which are prevalent in cross-organizational cybersecurity data. Without resolving this divergence, federated models may learn to suppress important features or exhibit inconsistent behavior across deployment sites—undermining their effectiveness and reliability. Recent work such as FedELC [9] addresses label noise in federated learning by identifying clients with corrupted annotations and refining their local labels through end-to-end correction. However, FedELC assumes a shared semantic interpretation of labels across participants. In contrast, the ILADE problem we address arises from systematic differences in labeling policies across organizations, where divergent yet valid interpretations lead to semantic inconsistencies. Unlike random noise, ILADE requires alignment of label meaning rather than correction of erroneous samples. Our method focuses on mitigating this semantic divergence through representation-level harmonization and alert filtering.

In this paper, we present an FL framework that addresses both the ILADE problem and model inversion risks through two key technical contributions:

- **Keyed Feature Hashing (KFH)**: We introduce a hashed representation layer that combines feature hashing [10] with key-dependent indexing under a single global key *k* shared by the SOC server and enrolled clients [11]. To the best of our knowledge, this is the first study to employ a keyed hash to seed feature hashing for cross-entity alignment in federated learning across organizations. KFH yields consistent cross-entity vectors while limiting raw-feature exposure and preventing index-probing enumeration; it is a lossy, many-to-one encoding rather than a standalone cryptographic safeguard.
- **Alert Filtering Mechanism**: Leveraging the semantic consistency of KFH-encoded vectors, we introduce a clustering-based filtering process that identifies alerts with conflicting or ambiguous label semantics. These alerts are excluded from training to prevent the propagation of inconsistent supervision signals, thereby improving the stability and accuracy of the global model.

We validate our approach on a large-scale real-world dataset collected from 14 institutions managed by a centralized SOC. Experimental results show that our method improves alert classification performance—achieving up to 13.36% higher F1-score—while maintaining over 99% classification coverage. These results demonstrate that our method enhances the reliability and robustness of FL-based models in distributed, label-divergent environments.

The remainder of this paper is organized as follows: the next section reviews related work, followed by the problem formulation and motivation. We then present our proposed methods, experimental results, and conclude with a summary.

## Related work

Network-based intrusion detection systems (IDSs) are widely used to monitor network traffic and detect cyberattacks or suspicious activities. These systems—such as Snort, Suricata, and Zeek—have long played a central role in security operations [12–15]. However, they typically generate an overwhelming number of alerts, many of which are false positives, leading to operator fatigue and reduced monitoring effectiveness [16–19].

To address this challenge, various AI-based techniques have been proposed to automate the analysis and prioritization of IDS alerts. For example, deep learning and graph-based models have been employed to detect patterns in high-volume alert data [6], while unsupervised recurrent neural networks have been used to complement web application firewalls for detecting zero-day attacks [20]. Other works have explored embedding-based models and sequential modeling approaches for improved alert classification and event correlation [21–24].

A critical limitation in many real-world IDS datasets is the issue of inconsistent or inaccurate labeling. Such inconsistencies can degrade the performance of ML models by introducing noise during training, leading to misclassifications and increased false alarm rates. To mitigate this, several studies have investigated data auditing and preprocessing techniques to improve label quality and consistency, especially in collaborative security environments [6,8,25].

Federated learning (FL) has recently emerged as a promising approach for collaborative model training, allowing multiple entities to jointly learn a global model without sharing raw data. The canonical FL algorithm, FedAvg, aggregates local model updates from distributed clients to maintain data locality and communication efficiency [1]. However, FedAvg suffers when data distributions are highly heterogeneous. To address this, FedProx introduces a regularization term to stabilize model convergence in non-IID settings [2], while FedAdam employs adaptive learning rates to improve training stability [3]. Recent surveys highlight that decentralized FL architectures—where no central server is present—introduce new security and privacy challenges due to their peer-to-peer structure and complex trust assumptions [26]. Despite these advancements, challenges such as communication overhead, hyperparameter tuning, and privacy-related risks persist.

In the cybersecurity domain, FL has been applied to IDS scenarios to improve attack detection while limiting raw data exposure. One study [27] examines FL architectures tailored for anomaly detection in heterogeneous and resource-constrained environments, highlighting issues such as federated poisoning attacks and non-IID data handling. Alazab et al. [28] present a comprehensive review of FL applications in cybersecurity, covering attack detection, trust management, and model robustness. However, one underexplored vulnerability in FL is the potential for trained models to be leaked or misused. Since global models are distributed to multiple entities, adversaries may attempt model inversion or misuse trained representations if the model is compromised [29]. This motivates the need for input encoding techniques that reduce interpretability while preserving learning utility.

Recent studies have revisited the threat of model inversion in federated learning, where adversaries attempt to reconstruct sensitive training data from shared gradients. A recent work [30] introduces SPEAR, a gradient matching attack that accurately recovers training batches even under realistic FL conditions, such as small batch sizes and batch normalization. Their findings underscore the urgent need for obfuscation mechanisms beyond traditional differential privacy. In response, another study [31] proposes HyperFL, a novel framework that breaks the direct connection between shared model parameters and local private data by leveraging hypernetworks. Unlike prior works relying on secure aggregation or gradient noise, HyperFL generates client models via private embeddings and transmits only hypernetwork parameters. This design makes input recovery via gradient inversion significantly more difficult, offering an improved privacy-utility trade-off without compromising performance.

While recent work such as FedELC [9] has addressed the issue of noisy labels in federated learning by identifying and correcting mislabeled data samples within individual clients, this line of research primarily assumes that all participants share a consistent semantic interpretation of class labels. FedELC improves robustness against corrupted annotations by detecting noisy clients and refining their local datasets through end-to-end label correction. However, such approaches do not consider the more nuanced challenge of *semantic divergence* in labeling across organizations. In contrast, the ILADE problem tackled in this paper arises not from random noise or annotation errors within a single dataset, but from systematic differences in labeling policies among multiple institutions.

While Yang et al. [7] propose a hierarchical and multimodal approach for analyzing semantically rich IDS alerts, their method assumes access to consistent ground-truth labels within a single organization. In contrast, our work focuses on a federated setting where semantic label divergence emerges due to differing interpretations across organizations. This divergence can significantly hinder the effectiveness of collaborative alert classification systems, motivating the need for mechanisms that can detect and mitigate such inconsistencies.

To the best of our knowledge, however, the specific issue of inconsistent labeling across different entities in federated learning for IDS alert classification has not been explicitly studied. This paper addresses this gap by identifying the problem and proposing effective mitigation strategies tailored to real-world SOC environments.

In SOC deployments, federated learning requires a fixed-dimensional, cross-entity–aligned representation that avoids raw-token exchange. Common encoders (one-hot/TF–IDF, learned embeddings, random projections, and plain feature hashing) face practical drawbacks in this context: one-hot/TF–IDF and learned embeddings require a shared vocabulary or ongoing inventory maintenance; random projections preserve distances but do not by themselves guarantee stable token-level alignment; and plain (unkeyed) feature hashing can yield cross-site compatibility when parties share the same hash function and dimensionality, but the resulting mapping is fixed and publicly enumerable, which enables index-probing enumeration (trying candidate inputs and linking non-zero indices over time). In contrast, our Keyed Feature Hashing (KFH) introduces a single global key *k* into feature hashing to produce a deterministic, fixed-dimensional, cross-entity–aligned index space without vocabulary sharing, while preventing index-probing against a public, fixed mapping. Consequently, KFH provides robustness to model inversion by preventing index-probing enumeration and achieves cross-entity alignment without any exchange of raw tokens or vocabularies.

## Problem and motivation

We clarify the scope upfront. Our primary objective is to mitigate ILADE—semantic inconsistency of labels across organizations. At the same time, we consider model exposure as a practical constraint of FL deployments. To address both, we use KFH to obtain a keyed, cross-entity–aligned representation (deployment-driven enabling layer), and we design AFL to directly reduce the effect of ILADE via selective filtering.

This study addresses a critical and underexplored challenge in FL for distributed alert classification: *inconsistent labeling among different entities* (ILADE). While FL enables privacy-preserving collaboration by training models without sharing raw data, its effectiveness can be significantly degraded when participating organizations assign divergent labels to semantically similar alerts.

**Fig 1:**
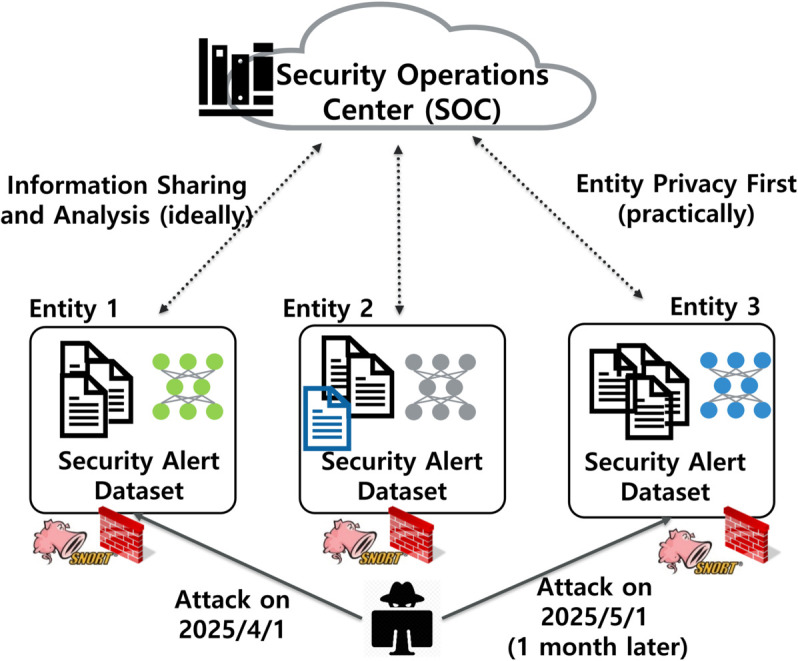
An example SOC managing multiple independent entities.

Each institution typically applies internal labeling criteria to IDS alerts, informed by local policies and operational context. For example, as shown in Fig 1, a centralized SOC may manage hundreds of independent entities, each using different interpretations for classifying alerts. If Entity 1 identifies a threat and labels it as an *Attack*, while Entity 3 later experiences a similar event but treats it as *Benign*, inconsistencies emerge in the federated training process. These inconsistencies introduce semantic conflicts that lead to noisy gradients and misaligned decision boundaries in the global model.

As IDS deployments generate large volumes of alerts—often over 10,000 per day per entity—manual triage becomes infeasible. According to [8], a human analyst can review only up to 76 alerts daily, underscoring the need for automated labeling models. Federated learning offers a way to build such models collaboratively while preserving data privacy. However, semantic label divergence like ILADE can substantially degrade FL performance.

While this paper focuses on network-level IDS alerts, which are widely used in operational security environments, the proposed methods are broadly applicable to any type of alert-based monitoring system, including host-based intrusion detection and suspicious activity reports. Our approach is model-agnostic and can be integrated into diverse alert processing pipelines where semantic inconsistencies in labeling are a concern.

### Inconsistent Labeling Among Different Entities (ILADE)

**Fig 2:**
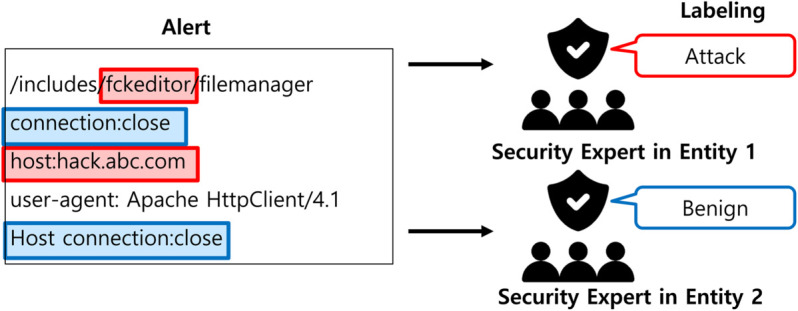
Label inconsistencies arising from different viewpoints. Label inconsistencies arise when entities interpret the same alert differently due to different viewpoints or security policies.

Semantic label divergence arises when similar alerts are assigned different classes due to differing institutional policies, security baselines, or usage contexts. As illustrated in Fig 2, an alert involving the XMRig cryptocurrency miner may be labeled *Attack* by one organization due to prior abuse in cryptojacking, but marked *Benign* by another that permits the tool for research purposes [32]. Such inconsistencies induce *conflicting gradient signals across clients* during aggregation, leading to noisy updates and degraded global model quality.

**Fig 3:**
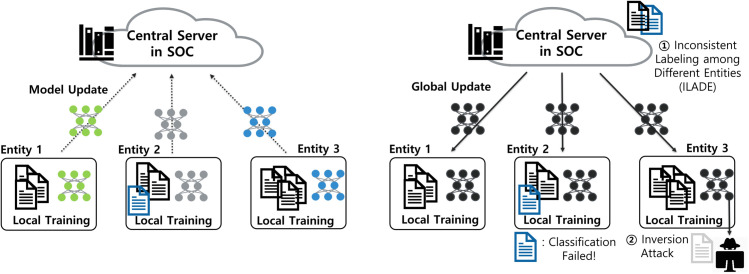
Schematic overview of federated learning in an SOC. The process involves local training, model update, and global aggregation, highlighting (1) inconsistent labeling among different entities (ILADE) and (2) model inversion risk.

Fig 3 provides a schematic overview of the federated workflow and the two deployment risks considered in this study—ILADE and model inversion—for workflow context. It also contrasts the ILADE phenomenon with the proposed AFL framework, which mitigates inter-label semantic divergence across entities. Details of the training protocol (communication rounds and early stopping) are provided in *FL Model Training*.

A similar divergence can occur with network scanning tools such as Nmap [33]. One institution may flag Nmap-generated traffic as *Attack* based on its use in reconnaissance phases of cyber intrusions, while another may label the same traffic as *Normal* if it originates from authorized internal vulnerability assessments. These inconsistencies confuse the FL optimization process, leading to conflicting updates and suboptimal convergence.

Unlike conventional FL challenges such as non-IID distributions or class imbalance, ILADE reflects a semantic-level misalignment. Addressing this issue requires techniques that go beyond reweighting or personalization and instead detect and isolate alert instances that are prone to disagreement. Our approach tackles ILADE by introducing a filtering mechanism that flags such alerts before they bias the model.

### Model inversion threats

In addition to label inconsistency, FL in cybersecurity contexts must address the risk of model exposure. Since the global model is distributed to multiple participating entities, there is an increased likelihood that it may be leaked or accessed by adversaries. If compromised, malicious participants or external attackers can attempt model inversion attacks to infer sensitive information about the training data [34,35]. This risk is particularly serious in security monitoring, where alerts may reveal organizational assets, internal network behaviors, or known threat signatures.

To mitigate this, we introduce KFH, a key-dependent obfuscated encoding scheme for alerts. KFH ensures that the vector representations used during FL training are both consistent across institutions and difficult to reverse-engineer without access to the shared key. This obfuscation reduces the risk of model inversion and supports collaborative learning across heterogeneous organizations by aligning feature representations without revealing raw alert content. Because the index mapping depends on the shared key *k*, the hashed vector is *not publicly enumerable*; in particular, KFH **prevents index-probing enumeration** that would be feasible under a fixed, unkeyed mapping (i.e., by trying candidate inputs and tracking non-zero indices).

## Adaptive federated learning for security centers

We propose a novel scheme, **Adaptive Federated Learning for Security Centers (AFL)**, to address two critical challenges in FL for IDS alert classification: the ILADE problem and model inversion attacks. AFL combines obfuscated encoding and a filtering mechanism to enhance model robustness and entity-specific accuracy.

**Fig 4:**
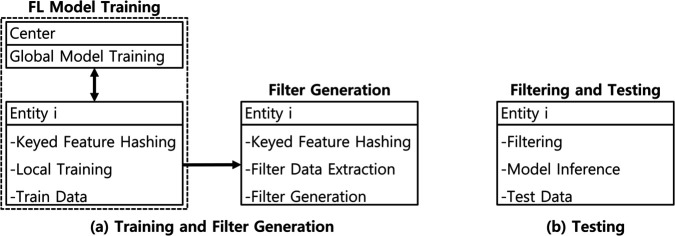
Training and testing phases of AFL.

The training and testing phases of AFL are illustrated in Fig 4. FL training is conducted collaboratively between the security center and its member entities. To defend against inversion attacks [36], we introduce KFH. Once a global model is obtained, each entity constructs a filter using local data to identify alerts likely to be misclassified due to ILADE.

During testing, each alert undergoes KFH encoding and is checked against the filter. Alerts flagged by the filter are excluded from automatic classification; others are labeled using the fine-tuned model. The following subsections detail each phase.

### FL model training

The first step of AFL is to train an FL model among the security center and member entities. Each entity splits its alert dataset into a training set and a filter-generation set. The training subset is used to build a local model whose parameters are sent to the central server. The server aggregates them into a global model, which is distributed back to the entities. This process repeats until convergence. The overall workflow is schematically summarized in Fig 3.

Although several improvements to FL such as FedProx [2] and FedAdam [3] have been proposed, we adopt FedAvg [1] due to its simplicity and comparable performance on our datasets.

To ensure interoperability in FL, all participating entities must use a consistent input vector format and model architecture [1]. To this end, we introduce KFH, a novel encoding scheme that combines feature hashing [10] with keyed hashing [11] to achieve consistent vector representations across entities while obfuscating raw alert content to mitigate model inversion risks.

For the model architecture, we adopt a lightweight feedforward neural network designed for binary classification. It comprises four fully connected layers, each followed by a ReLU activation function. The final output layer generates a single scalar value, which is passed through a sigmoid activation to produce a probability score.

**Fig 5:**
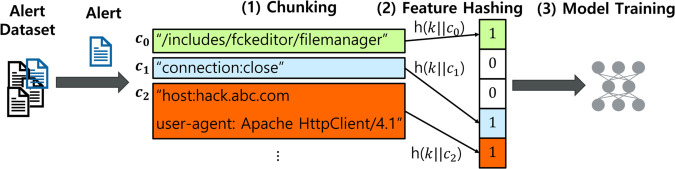
Keyed Feature Hashing (KFH) overview. KFH obfuscates alert data using a shared secret key among the security center and its participating entities, enabling consistent encoding across organizations and preventing index-probing enumeration that is feasible with fixed, unkeyed mappings.

Each alert is converted into a fixed-length vector, as illustrated in Fig 5. In this study, an alert refers to a byte sequence extracted from a packet, with a maximum length of approximately 1,500 bytes. To segment alerts into smaller components, we employ a content-defined chunking (CDC) algorithm [37,38]. Specifically, we adopt the AE chunking method with a default window size of four, yielding chunks with an average length of 6.88 bytes (i.e., 4×1.72), as depicted in Fig 5.

Each resulting chunk *c*_*i*_ is mapped to a position in an integer array *F*[*m*], initialized to zero, using a cryptographic hash function h(·) such that $F[h(c_{i})]:=F[h(c_{i})]$  +  1. This constitutes a simple feature hashing scheme, where $h(c_{i})\in \{0,1,...,m-1\}$ [10].

To enhance privacy, we extend this scheme with a keyed hashing mechanism. Each chunk is concatenated with a secret symmetric key *k* to form *k*||*c*_*i*_, and the hash computation becomes $F[h(k||c_{i})]:=F[h(k||c_{i})]$  +  1. As a result, no meaningful information about *c*_*i*_ can be inferred without knowledge of *k*.

We refer to this encoding scheme as KFH. By obfuscating feature representations, KFH enhances the robustness of the FL model against inversion attacks. The symmetric key *k* is assumed to be securely shared between the security center and its participating entities. Without access to *k*, it is extremely difficult for an adversary to reconstruct meaningful information from the input vectors, and even more so from the trained model.

**Fig 6:**
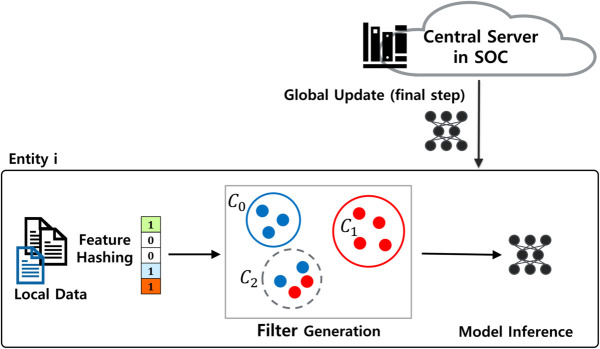
AFL incorporating a filtering mechanism to address semantic label divergence.

After the global FL model is trained and distributed, each participating entity evaluates its performance using a local training dataset. Due to the ILADE problem, the global model may still misclassify alerts that reflect semantic disagreements across organizations. To mitigate this issue, we introduce a filtering mechanism that identifies and excludes alerts likely to be affected by such inconsistencies. This process is illustrated in Figs 4 and 6, and is described in detail in the next subsection.

### Filter generation

After obtaining a fine-tuned model, each entity generates a filter to identify alerts that are likely to be misclassified due to the ILADE problem. This process is denoted as “Filter Generation” in Figs 4 and 6.

Prior to FL model training, the entity splits its local dataset into two subsets: the *training dataset* for FL training and fine-tuning, and the *filter-generation dataset* for constructing the filter. Once fine-tuning is complete, the filter-generation dataset is used to identify alerts that the model is uncertain about.

**Fig 7:**
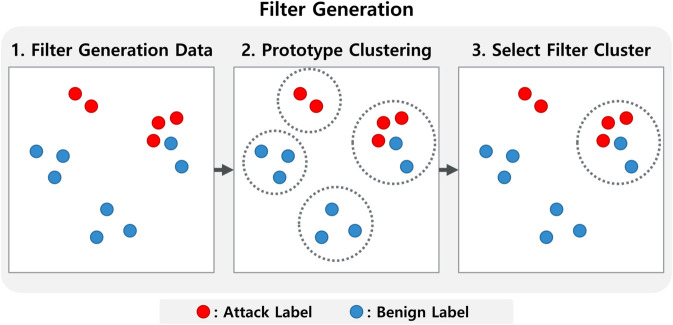
Filter generation process. The process uses obfuscated vectors from the KFH encoder to identify clusters prone to semantic label inconsistency.

To construct the filter, we apply a clustering algorithm to the obfuscated feature vectors produced by KFH encoder. For each alert in the filter-generation dataset, we compute its KFH-based representation, as illustrated in Fig 7. These vectors are then grouped via clustering to identify semantically inconsistent patterns.

We adopt a prototype clustering algorithm [39] due to its simplicity and efficiency, though other algorithms can be substituted. Clustering begins by selecting a seed vector; all vectors with cosine similarity above a threshold θ (set to 0.8) are grouped with the seed. The next seed is chosen as the vector least similar to the previous seed, and the process repeats until all vectors are assigned to clusters.

After clustering, we identify *confusing clusters*—clusters containing both *Attack* and *Benign* labels. These clusters are assumed to reflect decision boundaries where the model is unreliable. For each confusing cluster, we compute its centroid, which is used during testing to filter out ambiguous alerts.

For each cluster, let $n_{\text{Attack}}$ and $n_{\text{Benign}}$ denote the label counts and $n=n_{\text{Attack}}$  +  $n_{\text{Benign}}$. We define the label-mix ratio $r=\min\{n_{\text{Attack}},\;n_{\text{Benign}}\}/n$. A cluster is labeled *confusing* iff r≥τ (defaults τ=0.40).

### Filtering and testing

During the testing phase, each incoming alert is first evaluated against the entity’s filter. If the alert matches any filter, classification is skipped to avoid potential misclassification due to ILADE. Otherwise, the alert is classified using the global FL model. An alert is considered a match if the cosine similarity between its obfuscated vector (produced by the KFH encoder) and any cluster centroid exceeds the threshold θ.

We define *coverage* as the proportion of alerts that proceed to classification; for example, if 10 out of 100 alerts are filtered, coverage is 0.90. Coverage is standard in selective (abstaining) classification and has been widely adopted in prior work [40,41].

$$\text{Coverage}=\frac{\text{Number of non-filtered test alerts}}{\text{Total number of test alerts}}$$ (1)

### Evaluation metrics

We evaluate the performance of AFL using *precision*, *recall*, *F1-score*, and *coverage*. As described above, coverage is computed based on the number of alerts excluded by filtering. The remaining alerts are evaluated using standard classification metrics by counting the number of false positives (FP), true positives (TP), false negatives (FN), and true negatives (TN). Accordingly, AFL’s performance should be interpreted as F1 (or precision/recall) *together with* its *coverage*; reporting accuracy-like metrics without the corresponding coverage is not fair. The criteria are defined as follows:

- A **Benign** alert is a **false positive (FP)** if incorrectly labeled as **Attack**.
- A **Benign** alert is a **true negative (TN)** if correctly labeled.
- An **Attack** alert is a **true positive (TP)** if correctly labeled.
- An **Attack** alert is a **false negative (FN)** if incorrectly labeled as **Benign**.

The metrics are calculated as:

$$\text{Precision}=\frac{TP}{TP+FP}$$ (2)

$$\text{Recall}=\frac{TP}{TP+FN}$$ (3)

$$\text{F1-score}=2\times \frac{\text{Precision}\times \text{Recall}}{\text{Precision}+\text{Recall}}$$ (4)

## Experiments and validation

We evaluate AFL on a large-scale real-world dataset collected from 14 organizations under a centralized security operations center (SOC). AFL consistently improves classification performance across entities, increasing the average F1-score from 0.952 to 0.960. In the entity most affected by labeling inconsistency, the F1-score rises from 0.816 to 0.925, demonstrating that AFL effectively mitigates the ILADE issue. AFL also maintains an average alert coverage of 0.99, enabling reliable automated classification for the vast majority of alerts without requiring manual intervention.

### Experimental datasets and environment

#### Dataset.

We utilize private alert datasets collected from 14 entities affiliated with a security operations center (SOC) in South Korea. The SOC provides managed security services to over 100 organizations, operating hundreds of network-based intrusion detection systems (IDSs) deployed at Internet gateways. Alerts are generated in real time and analyzed by SOC personnel. Due to differences in labeling practices and institutional policies, semantic inconsistencies (ILADE) are frequently observed in the dataset, as illustrated in Figs 1 and 2.

The alert data was collected over a period of four years (2017–2020). For experimentation, we filtered the data to include only IDS alerts related to web-based attacks, resulting in 952,289 alerts across 62 distinct types, including directory listing attempts, Apache Struts errors, and cryptomining behavior. Each alert was labeled as either *Attack* or *Benign* by the corresponding entity.

Each entity’s dataset is partitioned into three disjoint subsets: training, filter-generation, and testing, as shown in Fig 4. The global FL model is trained using the training set. Filters are constructed locally using the filter-generation set. The testing set is used to evaluate performance in terms of precision, recall, F1-score, and coverage (Table 1).

**Table 1:** Dataset statistics.

Entity	Training	Filter Generation	Testing	Total
1	20,676	6,892	6,892	34,460
2	18,801	6,267	6,268	31,336
3	348,219	116,073	116,073	580,365
4	8,051	2,684	2,684	13,419
5	35,772	11,924	11,924	59,620
6	4,921	1,640	1,641	8,202
7	2,923	974	975	4,872
8	49,629	16,543	16,544	82,716
9	24,877	8,293	8,293	41,463
10	4,972	1,658	1,658	8,288
11	3,070	1,023	1,024	5,117
12	2,565	855	855	4,275
13	39,571	13,191	13,191	65,953
14	7,321	2,441	2,441	12,203
Total	**571,368**	**190,458**	**190,463**	**952,289**

#### Experimental setup.

We employ a feedforward neural network with three hidden layers consisting of 512, 256, and 128 nodes. The 768-dimensional input vector is generated using KFH. A sigmoid activation function is applied at the output layer to perform binary classification between *Attack* and *Benign*. The filtering and clustering processes are conducted directly on the obfuscated input vectors produced by the KFH encoder.

**Fig 8:**
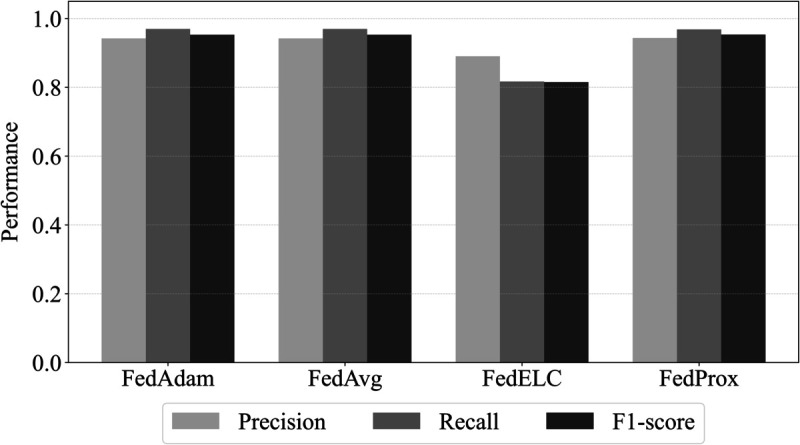
Comparison of representative FL optimizers. The figure compares FedAvg, FedProx, FedAdam, and FedELC based on macro-averaged precision, recall, and F1-scores across 14 entities. FedAvg, FedProx, and FedAdam show similar performance, while FedELC is slightly lower. Given their comparable results, FedAvg was chosen as the baseline for its simplicity and stability.

We adopt FedAvg [1] as the baseline FL method. To validate this choice, we additionally evaluated FedProx [2], FedAdam [3], and FedELC [9] under the same architecture and preprocessing. As shown in Fig 8, the macro-averaged precision, recall, and F1-scores across 14 entities are nearly identical for FedAvg, FedProx, and FedAdam, while FedELC is slightly lower overall. Importantly, all optimizers converge to a similar level, and none resolves the ILADE issue (e.g., the average F1 for Entity 10 remains around 0.8 across optimizers). Given these comparable results, we select FedAvg as the baseline FL algorithm for subsequent AFL experiments, due to its simplicity, stability, and representativeness in federated learning research.

We conducted a grid search over batch size, learning rate, local training epochs, and the number of FL rounds. The primary selection criterion was validation macro-F1, with coverage and training time used to break ties. The final configuration in Table 2 (batch size = 2048, learning rate = 0.001, local epochs = 10, communication rounds = 50) achieved the best validation macro-F1, while also balancing coverage and training time. Early stopping was applied based on validation loss: training stops after 5 consecutive non-improving epochs (patience = 5), and the model parameters from the epoch with lowest validation loss were restored.

**Table 2:** Hyperparameter settings for AFL and FedAvg experiments.

Parameter	Value
Batch size	2048
Learning rate	0.001
Local epochs	10
Communication Rounds	50
Cosine similarity threshold (θ)	0.8
Confusing cluster threshold (τ)	0.4

For comparison purposes, we implement three different neural network models:

- LOCAL model: Each entity trains independently without collaboration. This model serves as a baseline to highlight the need for collaborative approaches such as FL and AFL in SOC environments.
- FL model: A global model is trained via FedAvg using all entities’ data, following the standard FL method.
- AFL model: The proposed scheme with local filtering based on clustering. Each entity applies clustering to its local data to generate entity-specific filters, which are then used to refine predictions from the global model. This constitutes the AFL scheme proposed in this paper.

Experiments run on an AMD Ryzen 9 7950X3D CPU 16-Core Processor, NVIDIA RTX 4090 GPU, 128GB of memory, and 4TB SSD storage, implemented in Python 3.9 with Scikit-learn 1.5.1 and PyTorch 2.3.1.

#### Global Test Dataset (Global Test) vs. Local Test Dataset (Local Test).

To highlight the advantage of FL in SOC settings, we construct a *global test dataset* by aggregating the test datasets from all participating entities. In contrast, the *local test dataset* refers to each entity’s own individual test set. Experimental results demonstrate that FL significantly outperforms LOCAL models when evaluated on the global test dataset, indicating superior generalization across institutional boundaries.

#### Multi-seed evaluation and variability analysis.

We repeat all main experiments with three independent random seeds for training and testing. Figures report the mean and standard deviation across three runs, where the whiskers represent the variability of results among seeds. Because the sample size is small (*n* = 3), we used standard deviation rather than confidence intervals to directly represent dispersion. This approach provides a more intuitive indication of variability without introducing additional assumptions about distributional properties. Across all runs, the relative ordering among methods and their high coverage remain consistent; thus, our conclusions are unaffected.

### Experimental results

#### Experiments on private dataset.

**Fig 9:**
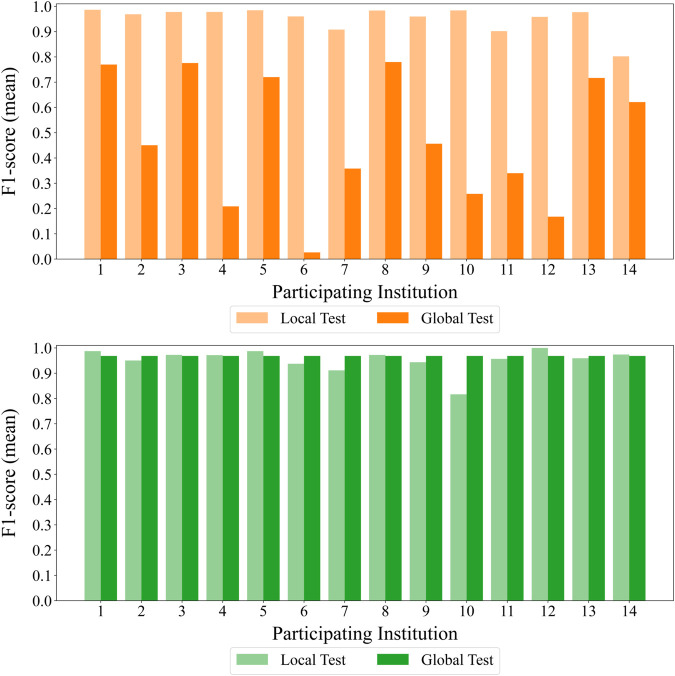
Comparison of LOCAL and FL models. The figure evaluates performance on local (left) and global (right) test datasets. Each bar indicates the mean F1-score over three runs. (A) LOCAL models generalize poorly across entities. (B) The FL model generalizes better overall but shows a sharp drop in Entity 10 due to semantic label divergence (ILADE).

We first compare the LOCAL and FL models using the private dataset. Both models are evaluated on the global test dataset as well as each entity’s local test dataset. As shown in Fig 9(A), LOCAL models perform well when tested on their corresponding local datasets but show substantial performance drops on the global test dataset. In this figure, the *x*-axis represents the entity ID, and the *y*-axis denotes the mean F1-score across three independent runs. This disparity suggests that LOCAL models tend to overfit to their specific environments and struggle to generalize across entities.

In contrast, the FL model achieves consistently high mean F1-scores on the global test dataset, as indicated by the dark green bars in Fig 9(B). This result highlights the value of FL in achieving robust, generalized performance in SOC environments.

However, despite its improved generalization, the FL model also reveals limitations. When evaluated on the local test datasets, it underperforms notably in Entity 10, as shown by the light green bar in Fig 9(B). This performance drop is attributed to the ILADE problem, where inconsistencies in labeling across entities hinder the model’s ability to adapt to localized decision criteria.

**Fig 10:**
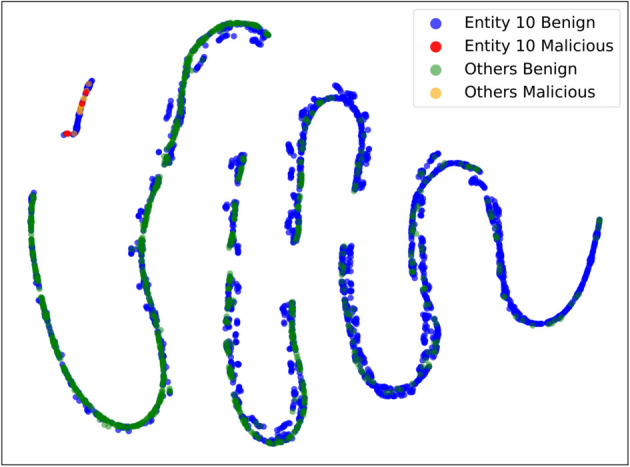
t–SNE visualization of latent representations. Benign (blue) and malicious (red) flows from Entity 10 are clustered in the same region as malicious flows (orange) from other entities, illustrating semantic label divergence (ILADE).

To further illustrate the performance degradation observed in Entity 10, we visualize its latent feature space using t–SNE (Fig 10). Unlike other entities, where benign and attack samples form clearly separated clusters, Entity 10 exhibits substantial overlap between benign and attack flows. Notably, many of its samples lie within the region occupied by malicious flows from other entities. This provides direct evidence of semantic label divergence (ILADE), where identical or similar flows are inconsistently labeled across entities. Such inconsistency accounts for the F1–score drop of Entity 10 under the FL model and underscores the importance of AFL’s filtering mechanism in mitigating this issue.

**Fig 11:**
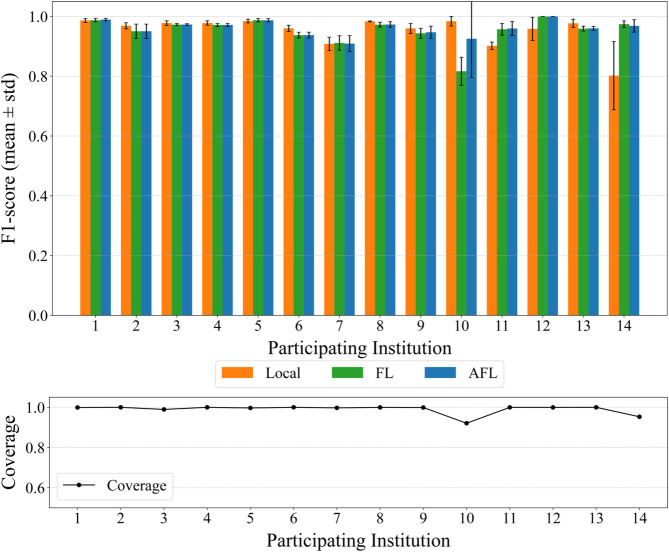
Local test results. Results obtained when θ=0.8 and τ=0.4. (A) Per-entity F1-scores (mean ± standard deviation over three runs) on local test sets. AFL shows comparable or higher performance than FL for several entities, while maintaining stable results under ILADE conditions. (B) Per-entity coverage (mean over three runs) on local test sets. AFL maintains consistently high coverage across institutions.

**Fig 12:**
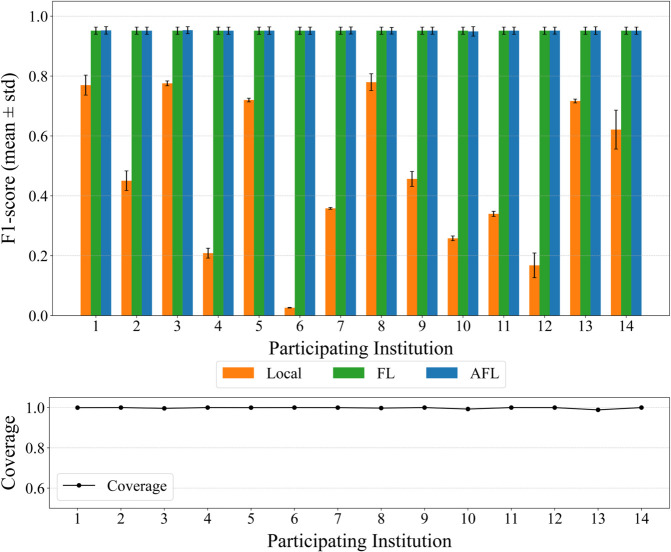
Global test results. Results obtained when θ=0.8 and τ=0.4. (A) Per-entity F1-scores (mean ± standard deviation over three runs) on the global test set. AFL maintains higher or comparable global performance compared to FL and LOCAL across entities, demonstrating stable global generalization. (B) Per-entity coverage (mean over three runs) on the global test set. AFL sustains near-complete coverage.

In the second set of experiments, we evaluate the proposed AFL model on the same dataset. The model is assessed using both the global test dataset and each entity’s local test dataset. As shown in Figs 11 and 12, AFL demonstrates stable and generally improved performance across both evaluation settings. These results indicate that AFL can enable near-complete automation in alert labeling by providing both high accuracy and stable coverage.

Designed to mitigate the ILADE problem, AFL alleviates the limitations observed in both LOCAL and FL models. Experimental results confirm that it consistently outperforms both baselines, particularly in the local test setting.

Fig 11(A) presents the per-entity F1-scores of LOCAL, FL, and AFL models evaluated on their respective local test data. Bars indicate the mean ± standard deviation across three independent runs. AFL shows higher or comparable F1 performance across most entities, while maintaining stable results even in Entity 10, where performance variation was previously observed. This improvement is attributed to AFL’s integration of filtering mechanisms with local clustering, which enables entity-specific adaptation while maintaining generalizability. Fig 11(B) further shows that AFL maintains high coverage across entities, complementing its accuracy.

Similarly, Fig 12(A) reports per-entity F1-scores on the global test set, where AFL and FL exhibit similar mean performance across most entities (whiskers denote ±1 sd over three seeds). Fig 12(B) shows that AFL sustains near-complete coverage on the global test. The per-entity gains at ILADE-affected sites are observed in the earlier local-test plot (Fig 11(B)); for example, Entity 10 improves from ≈0.8 (FL) to >0.9 (AFL). Taken together, these results underscore AFL’s robustness at ILADE sites, and we do not assert an overall performance increase over FL.

To further examine how performance varies with the filtering parameters, we summarize results for θ∈{0.6,0.7,0.8} and τ∈{0.2,0.3,0.4} in Table 3. The table reports mean ± standard deviation of precision, recall, F1, and coverage over three runs. Across all parameterizations, AFL maintains consistently high coverage (approximately 0.99) with only marginal fluctuations, and its F1 remains stable—continuing to surpass LOCAL and slightly exceed FL.

**Table 3:** Performance summary across different sweep settings of θ and τ. Values are reported as mean ± std over three runs.

θ	τ	Precision	Recall	F1	Coverage
0.7	0.2	0.880 ± 0.155	0.923 ± 0.132	0.898 ± 0.140	0.922 ± 0.086
0.7	0.3	0.903 ± 0.142	0.939 ± 0.128	0.919 ± 0.131	0.952 ± 0.072
0.7	0.4	0.935 ± 0.084	0.976 ± 0.019	0.953 ± 0.049	0.982 ± 0.066
0.8	0.2	0.867 ± 0.151	0.909 ± 0.138	0.886 ± 0.142	0.955 ± 0.060
0.8	0.3	0.939 ± 0.050	0.970 ± 0.031	0.952 ± 0.034	0.986 ± 0.021
0.8	0.4	0.947 ± 0.044	0.977 ± 0.018	0.960 ± 0.026	0.989 ± 0.012
0.9	0.2	0.877 ± 0.160	0.905 ± 0.153	0.888 ± 0.155	0.972 ± 0.037
0.9	0.3	0.940 ± 0.056	0.960 ± 0.039	0.949 ± 0.046	0.990 ± 0.017
0.9	0.4	0.947 ± 0.048	0.971 ± 0.028	0.958 ± 0.038	0.996 ± 0.011

This indicates that AFL’s gains do not rely on aggressive data removal but rather on selectively abstaining on a small set of uncertain samples. The similarity of trends across parameter settings suggests that, for this dataset, cluster geometry around decision boundaries is relatively simple, so moderate relaxations or tightenings of θ (centroid-match strictness) and τ (confusing-cluster criterion) alter the filtered subset only at the margins, leaving both coverage and F1 largely unchanged.

### Discussion

This study used alarm data from network-based IDSs. Each alarm contains anonymized attack names, network packet payloads (up to 1600 bytes), timestamps, and institution IDs. Due to data access limitations, endpoint detection and response (EDR) alarms could not be included [6–8]. However, the proposed methods are expected to be applicable to other types of alarms generated in security operations.

Our threat model assumes a semi-trusted consortium—an SOC-operated server and vetted participating institutions [5,8], rather than an open set of untrusted individuals. Under this assumption, a global *k* is necessary for consistent hashing and effective aggregation. We acknowledge that if fully malicious clients are present, sharing the same k does not by itself preclude all attacks; this is a limitation of our setting.

Recent advancements in large language models (LLMs) have prompted interest in applying them to various domains. This study attempted to vectorize alarm data using a BERT tokenizer for federated learning. However, the unstructured nature of network attack alarms posed significant challenges for effective tokenization, resulting in low F1-scores. Fine-tuning open-source LLMs on security monitoring datasets is suggested as a topic for future research.

## Conclusion

This study explored two critical obstacles to collaborative machine learning in real-world security monitoring environments: semantic inconsistencies in alert labels across institutions and the risk of sensitive information leakage through model inversion. To address these challenges, we proposed an adaptive federated learning framework that integrates a filtering mechanism and a keyed feature hashing technique. This design enables robust model training across distributed organizations while obfuscating input representations to safeguard against inversion attacks.

Our method was validated using a large-scale dataset collected from 14 institutions operating under a centralized security operations center. Experimental results demonstrated significant improvements in classification accuracy and alert coverage, particularly in the presence of inter-organizational label inconsistencies.

By supporting joint model development without raw data sharing or vulnerability to inference attacks, the proposed approach contributes to the design of secure, interpretable, and practically deployable machine learning systems. These findings are especially relevant to domains such as finance and critical infrastructure, where privacy, accuracy, and trust are essential to collaborative information processing.

## Code availability

The source code for the proposed method in this study is publicly available at the following GitHub repository: https://github.com/kmuinfosec/AFL-Mitigating-Semantic-Label-Divergence-in-Federated-Learning.
